# Dietary Fat Intake and Metabolic Syndrome in Older Adults

**DOI:** 10.3390/nu11081901

**Published:** 2019-08-14

**Authors:** Alicia Julibert, Maria del Mar Bibiloni, David Mateos, Escarlata Angullo, Josep A. Tur

**Affiliations:** 1 Research Group on Community Nutrition & Oxidative Stress, University of Balearic Islands, IDISBA & CIBEROBN, 07122 Palma de Mallorca, Spain; 2 Escola Graduada Primary Health Care Center, IBSalut, 07001 Palma de Mallorca, Spain

**Keywords:** older adults, macronutrient intake, dietary intake, fat intake, metabolic syndrome

## Abstract

Background: Metabolic Syndrome (MetS) is associated with higher rates of cardiovascular disease (CVD), type 2 diabetes mellitus, and cancer worldwide. Objective: To assess fat intake in older adults with or without MetS. Design: Cross-sectional nutritional survey in older adults living in the Balearic Islands (*n* = 477, 48% women, 55–80 years old) with no previous CVD. Methods: Assessment of fat (total fat, MUFA, PUFA, SFA, TFA, linoleic acid, α-linolenic acid, marine and non-marine ω-3 FA, animal fat and vegetable fat, cholesterol) and macronutrient intake using a validated food frequency questionnaire, and its comparison with recommendations of the US Institute of Medicine (IOM) and the Spanish Society of Community Nutrition (SENC). Results: Participants with MetS showed higher BMI, lower physical activity, higher total fat and MUFA intake, and lower intake of energy, carbohydrates, and fiber than participants without MetS. Men and women with MetS were below the Acceptable Macronutrient Distribution Range (AMDR) proposed by IOM for carbohydrates and above the AMDR for total fat and MUFAs, and women were below the AMDR proposed for α-linolenic acid (ALA) compared with participants without MetS. Conclusions: Subjects with MetS were less likely to meet IOM and SENC recommendations for fat and macronutrient intakes as compared to non-MetS subjects.

## 1. Introduction

Metabolic syndrome (MetS) is a clinical condition characterized by several metabolic risk factors [1,2] associated with higher prevalence of cardiovascular disease (CVD), type 2 diabetes (T2DM), and cancer worldwide [3]. These factors involve abdominal obesity, blood pressure, glycaemia, triglyceridemia (TG), and high-density lipoprotein cholesterol (HDL-c) [1].

The prevalence of MetS has been increasing over the years and is now reaching epidemic proportions [4]. In Western countries, the prevalence of MetS is approximately one-fifth of the adult population and increases with age. However, the prevalence of MetS will vary according to the population studied, age, gender, race, and ethnicity, as well as the definition applied [5,6]. 

MetS is also influenced by nutrient intake, alcohol consumption, physical exercise, or smoking [3]. Unhealthy eating patterns and lifestyle, such as malnutrition and inactivity, can worsen the clinical status, with accumulation of body fat and alteration of the parameters that characterize MetS [7]. 

As shown in the ANIBES study, the macronutrient distribution is worsening and somewhat moving away from the recommendations and traditional Mediterranean dietary pattern, although the negative changes are less pronounced as age increases [8]. Age, sex, lower levels of education, economic status, smoking status, and alcohol intake predict lower dietary variety. There is evidence that older Spanish adults with MetS had a high risk of inadequate nutrient intake [9].

Eating patterns and their food and nutrient characteristics are the primary emphasis of the recommendations of U.S. Dietary Guidelines 2015–2020 [10]. Accordingly, therehas been a focus on the roles of macronutrients (carbohydrates, fat, and proteins) [11,12,13,14,15,16,17] and dietary patterns [7,18,19,20] on MetS. 

Therefore, taking into consideration the scientific evidence on nutrients in the development of MetS, this study aimed to assess fat intake in older adults with or without MetS.

## 2. Materials and Methods 

### 2.1. Design and Participants

The sample had477 participants (48% women; aged 55–80 years old)with no previously documented CVD that were engaged in social and municipal clubs, health centers, and sport clubs ofacross-sectional study conducted in the Balearic Islands. The age range was chosen since they are at high risk of suffering non communicable disease, the association of MetS with CVD, and because the increasing prevalence of MetS with age is known [21]. Exclusion criteria included being institutionalized, suffering from a physical or mental illness thatlimited their participation in physical fitness or their ability to respond to questionnaires, chronic alcoholism or drug addiction, and intake of drugs for clinical research over the past year.

The study protocols followed the Declaration of Helsinki ethical standards, and were approved by the Ethics Committee of Research of Balearic Islands (refs. CEIC-IB2251/14PI and CEIC-IB1295/09PI). All participants provided informed written consent. 

### 2.2. Anthropometric Measurements

Anthropometric variables were measured by trained personnel to minimize the inter-observer coefficients of variation. Weight and height were measured with high-quality electronic calibrated scales and a wall-mounted stadiometer, respectively. Height was determined using a mobile anthropometer (Seca 213, SECA Deutschland, Hamburg, Germany) to the nearest millimeter, with the participant’s head maintained in the Frankfort Horizontal Plane position. Body weight and body fat were determined using a Segmental Body Composition Analyzer (Tanita BC-418, Tanita, Tokyo, Japan). The participants were weighed in bare feet and light clothes (0.6 kg was subtracted for their clothing). Body mass index (BMI) was calculated as weight in kilograms divided by the square of height in meters (kg/m^2^). Waist circumference (WC) was measured half-way between the last rib and the iliac crest by using an anthropometric tape. Blood pressure was measured using a validated semi-automatic oscillometer (Omron HEM-705CP, Hoofddorp, The Netherlands) after 5 min of rest inbetween measurements while the participant was in a seated position. All anthropometric variables were determined in duplicate, except for blood pressure (in triplicate).

### 2.3. Blood Collection and Analysis

Blood samples were collected after an overnight fast and biochemical analyses were performed on fasting plasma glucose, total cholesterol, HDL-c, and TG concentrations in local laboratories using standard enzymatic methods. Participants were classified as “with MetS” (*n* = 333) and “without MetS” (*n* = 144) according to the updated harmonized definition of the International Diabetes Federation and the American Heart Association and National Heart, Lung, and Blood Institute [2].

### 2.4. Dietary Intake Assessment

Licensed dieticians administered a semiquantitative, 137-item food frequency questionnaire (FFQ), repeatedly validated in Spain [22]. For each item, a typical portion size was included and consumption frequencies were registered in 9 categories that ranged from “never or almost never” to “≥6 times/day”. Energy and nutrient intakes were calculated as frequency multiplied by nutrient composition of specified portion size for each food item, using a self-made computerized program based on available information in the Spanish food composition tables by Moreiras et al. [23]. When foods in the Spanish food composition tables were not available, the BEDCA food database was used in order to complete missing information [24]. Dietary intake of energy, carbohydrates (CHOs), proteins, total fat, monounsaturated fatty acids (MUFAs), polyunsaturatedfatty acids (PUFAs) and SFAs, trans-fatty acid (TFA), linoleic acid (LA), α-linolenic acid (ALA), marine and non-marine ω-3 fatty acid (ω-3 FA), animal fat and vegetable fat, cholesterol, and fiber were estimated. The vegetable fat included vegetables, fruits, nuts, legumes, total cereals, olives, oils, cookies, fritters, cocoa powder, mustard, ketchup, fried tomato, sugar, marmalade, and snacks. The animal fat included total dairy products, total meat, total fish, pizza, butter, lard, bakery goods, nougat, ready-to-eat meals, salad cream, and honey. The fat quality index (FQI) was also calculated as previously described [25]. Briefly, the FQI was calculated using the ratio (MUFA + PUFA)/(SFA + TFA) as a continuous variable.

Macronutrients and different fat intakes were compared with Institute of Medicine (IOM) and Spanish Society of Community Nutrition (SENC) recommendations. The dietary references intakes (DRIs) values proposed by IOM [26] were used, which are quantitative estimates of nutrient intakes to assess and plan diets for healthy people, including the Acceptable Macronutrient Distribution Range (AMDR) values. The prevalence of inadequate macronutrient intake according to the 2020 Nutritional Objectives for Spanish Population proposed by SENC [27] was used.

### 2.5. Socioeconomic and Lifestyle Determinants

Sociodemographic and lifestyle characteristics were collected from each participant. Educational level was ranked into primary school, secondary school, and university. Physical activity was measured using the validated Spanish version of the Minnesota Leisure Time Physical Activity Questionnaire [28,29]; it was taken by interview with trained research assistants and measured leisure time physical activities (LTPA), including household activities, over the previous 12 months. The Minnesota questionnaire was used to estimate physical activity levels by using metabolic equivalents of tasks (METs) [30]. METs are calculated by multiplying the intensity (showed by the MET-score) and the duration spent on that activity (measured in minutes). The MET-score can be derived from tables (the Compendium of Physical Activities) [31] that show the intensity of the activity relative to resting (METhours/week) spent on physical activity refer to the energy that is spent on activities, over and above existing levels of resting energy expenditure. Finally, information related to individual medical history, current medication use, and smoking status were also obtained.

### 2.6. Statistical Analyses

Analyses were performed with the SPSS statistical software package version 25.0 (SPSS Inc., Chicago, IL, USA). All analyses were stratified by sex and MetS status. Data are shown as mean, standard deviation (SD), or median and interquartile range (IQR). Normality of data was assessed using Kolmogorov–Smirnov test. Difference in medians between two comparison groups were tested by the Mann-Whitney U-test when variables were not normally distributed, and difference in means between the two comparison groups were tested by unpaired Students’ *t*-test when variables were normally distributed. Differences in prevalence of MetS or not among participants were examined using χ^2^ (all *p* values are two-tailed). Logistic regression analyses with the calculation of corresponding odds ratio (OR) and the 95% confidence interval (95% Confidence Interval, CI) were also used to assess the association between pathological features of MetS and macronutrients, specific types of fat, and dietary intake. Results were adjusted for sex, age (continuous variable), BMI (continuous variable), energy intake (continuous variable), and total physical activity (continuous variable, expressed as METmin/hour) to control for potential confounders. Results were considered statistically significant if *p*-value (2 tailed) <0.05. 

## 3. Results

Comparison of socioeconomic and lifestyle characteristics between the two study groups stratified by sex are shown in Table 1. Participants with MetS showed higher BMI and lower total physical activity than participants without MetS. As expected, the groups differed in all MetS components, except for blood pressure in women. A higher percentage of patients with MetS showed pathological cut-off values than patients without MetS in all MetS components.

Male MetS patients with high blood pressure plus hyperglycemia plus high abdominal fat comprised 64.5% of the total MetS population; those with high blood pressure plus hypertriglyceridemia plus low HDL-c comprised 42.6% of the MetS population. Female MetS patients with high blood pressure plus hyperglycemia plus high abdominal fat comprised 59.3% of the total MetS population; those with high blood pressure plus hypertriglyceridemia plus low HDL-c comprised 38.7% of the MetS population.

Comparisons of nutrient intakes and food consumption between the two study groups stratified by sex are shown in Table 2 and Table 3, respectively. Participants with MetS showed higher total fat and MUFA intake but lower intake of energy, carbohydrates, and fiber than those without MetS (*p* < 0.05). Participants with MetS also showed higher FQI than non-MetS participants. Women with MetS reported higher intake of proteins but lower intake of TFA, ω-3 FA, LA, ALA, and marine and non-marine ω-3 FA than women without MetS. Participants with MetS reported lower consumption of fruits, potatoes, total cereals, whole grain bread, and rice and pasta than participants without MetS. Men with MetS reported lower consumption of ready to-eat-meals than those without MetS. On the other hand, women reported lower consumption of bakery goods and alcohol than those without MetS.

Table 4 shows that participants with MetS, for both men and women, were more likely to be below the AMDR proposed by IOM for carbohydrates and ALA (except for men) and more likely to be above the AMDR for total fat and MUFAs than participants without MetS. Similar results were obtained when the 2020 Nutritional Objectives for the Spanish population were assessed (Table 5). Participants with MetS were also more likely to be below the acceptable nutritional range for carbohydrates and more likely to be above the acceptable nutritional range for total fat and MUFAs than participants without MetS. Finally, participants with MetS were more likely to be below the 2020 Nutritional Objectives for the Spanish population for TFA but also for total fiber, such as in fruits and vegetables.

Multivariate adjusted odds ratio (OR) for the association between pathological features of the MetS components and dietary macronutrient intake in participants with and without MetS showed, after adjustment for potential confounders (i.e., age, sex, BMI, energy and physical activity), that hypertension (equal or higher pathological cut-off value was OR reference: 1.00) is related with lower intake of PUFA (OR: 0.95; 95% CI: 0.91–0.98), SFA (OR: 0.95; 95% CI: 0.92–0.99), TFA (OR: 0.95; 95% CI: 0.91–0.99), LA (OR: 0.94; 95% CI: 0.90–0.98), and ALA (OR: 0.95; 95% CI: 0.91–0.99). However, abdominal obesity (equal or higher pathological cut-off value was OR reference: 1.00) was associated with high PUFA intake (OR: 1.10; 95% CI: 1.01–1.19), LA (OR: 0.12; 95% CI: 1.02–1.23) and vegetable fat (OR: 1.05; 95% CI: 1.01–1.08). No other relationships were found between other pathological components of MetS and dietary macronutrient intake.

## 4. Discussion

Subjects with MetS and without MetS showed differences for energy and macronutrient intake, as well as for intake of specific fat subtypes.

Energy and nutrient intake in MetS subjects revealed a diet lower in calories and carbohydrates, but higher in total fat and MUFA than those without MetS. Carbohydrate intake of MetS subjects was below the recommended limits (45–65% of total energy intake) and total fat intake of the same subjects was above the recommended limits (20–35% of total energy intake). Women with MetS showed more energy intake from protein than those without MetS (18% vs. 16.9%, respectively) (*p* < 0.01), but both were within recommended ranges [26,27]. A similar nutrient distribution among Spanish population with MetS [32] and healthy adults has been previously shown [8]. Differences were also previously observed between subjects with and without MetS for total energy intake, sugar intake, dietary glycemic load, percentage of dietary protein, PUFA, and fiber intake [33]. 

Despite women with MetS reporting lower consumption of bakery goods than those without MetS, differences in sugary food intake (bakery goods, dairy desserts, beverages, fruit juices, breakfast cereals, marmalade, ice creams, chocolate, and ready-to-eat meals) between subjects with and without MetS were not found in our study when the 2020 Nutritional Objectives for the Spanish population were assessed. Total sugar intake was also quantified in the ANIBES study: results were higher in children (17.18%) and adolescents (16.33%) and markedly lower in adults (15.34%) and older adults (12.97%) [8]. The inhabitants of Northern Spain, especially men, consumed more sugar and sweets than adult from other Spanish areas [32]. Conversely, the World Health Organization (WHO) recommended <10% of energy intake be provided by sugars [34], whereas <5% has been recommended in the United Kingdom [35]. It is well known that simple sugar intake is associated with significantly higher risk of developing MetS, including increased blood pressure, central obesity, and serum TG and glucose levels [36,37,38]. Frequent consumption of sugar-containing foods can also increase the risk of dental caries [39].

This study also demonstrated an association of gender and fat intake for MetS risk. Women showed an inverse association between fat intake and MetS, irrespective of fatty acid type. Women consumed less ω-3 and ω-6 FA, which could be related to the lower consumption of nuts observed in this group. Previously, Bibiloni et al. [40] showed that nut consumers were less likely to be below the estimated average requirement (EAR) for some nutrients and above the adequate intake (AI) for others than non-nut consumers. Other studies showed that European Food Safety Authority (EFSA) recommendations for intake of different types of ω-3 and ω-6 FA, such as LA, ALA, and eicosapentaenoic acid (EPA) + DHA, were not met in around half, one-quarter, and three-quarters of the European countries, respectively [41]. The most recent reviews also concluded that in half of the countries worldwide, the reported average PUFA intake was lower than the recommended range of 6–11% of energy [42,43,44]. In addition, the ω-3 and ω-6 FA intake was inversely associated with MetS prevalence in females [45]. In our study, total PUFA and specific types of PUFA (LA or ALA) intake were inversely associated with high blood pressure and positively associated with abdominal obesity. Evidence from observational and intervention studies supports the benefits of both ω-3 and ω-6 PUFA in reducing MetS [37,46,47,48,49], although other studies showed conflicting results [49,50,51]. Particularly, the adequate intake of MUFA and PUFAs in the PREvención con DIeta MEDiterránea (PREDIMED) study, mainly due to a high consumption of nuts and olive oil, has been previously associated with better adherence to the Mediterranean diet (MedDiet) [40] and to lower risk of CVD [52]. Moreover, other dietary patterns (Dietary Approaches to Stop Hypertension (DASH), new Nordic and vegetarian diets) have also been proposed as alternatives to the MedDiet for preventing MetS [5].

It is also worth noting that no differences were observed between subjects with and without MetS for SFA and animal fat, although participants without MetS showed higher consumption of bakery goods than those with MetS. Moreover, an association between pathological features of MetS and dietary macronutrient intake showed that hypertensionwas inversely associated with SFA. Contrarily to our results, a positive association between SFA intake and MetS components has been observed in most studies [46,50,53,54,55,56], although other studies pointed to a lack of association [49,57]. On the other hand, increased vegetable fat intake was positively associated with abdominal obesity; certain vegetable products may also have high saturated fat contents, such as coconut oil and palm kernel oil, along withmany prepared foods [10,58]. Moreover, most of the countries reported an average higher SFA intake than the recommended maximum of 10% of energy [42,43,44]. A prospective study with an older adult population at high risk of cardiovascular disease also observed an average higher SFA intake (10.3%) [59]. However, there is evidence that the intake of these fats is lower in the adults and older adults in the Mediterranean population, who consume low amounts of processed food; olive oil and meat ranked as the primary individual contributors [8].

Moreover, our findings show that women with MetS consumed more energy from TFA than those without MetS (6.4% versus 7.8%, respectively) (*p* < 0.005). Accordingly, TFA intake was inversely associated with hypertension. In a previous study, plasma TFA concentrations were significantly associated with MetS prevalence and its individual components, except for blood pressure [60]. In another study, the reduction in TFA intake over 1 year was significantly associated with a reduction in low-density lipoprotein particle number (LDL-P), a novel marker of CVD risk [61]. Actually, the 2015–2020 U.S. Dietary Guidelines for Americans and the IOM both recommend that individuals should limit TFA intake as much as possible to avoid their adverse effects on health [62]. 

Otherwise, the current findings showed that participants with MetS consumed less dietary fiber than the recommended dietary allowances (35 g for males and 25 g for females of this age group), which may be linked to low consumption of fruits and vegetables in our population study. This outcome is according tothe outcomes of a previous meta-analysis that provided a potential link between dietary fiber consumption and MetS risk factors [63]. Previous studies also showed a protective effect of fruit intake on MetS development [17,64,65,66], as well as a protective role on CVD development [67].

Finally, our results also show higher BMI and lower total physical activity in participants with MetS (*p* < 0.001), which is in agreement with a previous study that also showed higher level of physical activity in the control group compared to the MetS group, although this difference disappeared when the subjects were separated by sex and adjusted for total energy intake [16]. Another previous study showed that participants with lower levels of physical activity, being overweight and obese, were associated with higher risk of CVD. Accordingly, the impact of physical activity on CVD might outweigh that of BMI among middle-aged and elderly participants [68]. There is evidence that interventions including regular physical activity practice in patients with MetS improves MetS risk factors [69,70,71,72,73,74,75], indicating that maintaining a good physical condition would be essential for a healthy status.

### Strengths and Limitations of the Study

This study has several strengths. First, to our knowledge our study provides data on the intake of macronutrients and different types of fat in older adults with MetS or without it, which has been scarcelyreported previously. Our research also provides information about dietary fat intake in comparison to national and international recommendations, which may provide references for future public policies.

Some methodological limitations should be acknowledged. First, the cross-sectional study nature; thus, causal inferences cannot be drawn. Second, the relatively small sample size, specifically in the non-MetS group; for this reason, these findings cannot be generalized to the broader community based on this study alone. Third, the FFQ, the source of information to assess dietary fat intake, could overestimate the intake of certain food groups, even thosethat have been validated. In our study, a trained dietician conducted the interviews to collect the food frequency data; it is hoped that this approach (as compared with self-administration) reduced any potential misclassification bias. Another limitation of this study was that the used food composition databases showed missing or uncalculated data forseveral fats and fatty acid contents; these missing data are lower than 5% of all analyzed foods (for total fat, SFA, MUFA, PUFA, and cholesterol contents) and lower than 10% of foods (LA, ALA, trans-fat, EPA, DHA, and DPA are mainly from marine species and may change according to season, source, such as wild or from a fish farm, and cooking method) [76].

## 5. Conclusions

Subjects with MetS were less likely to meet IOM and SENC recommendations for fat and macronutrient intake as compared to non-MetS subjects. A healthy lifestyle is critical to prevent or delay the onset of MetS in older adults and to prevent CVD in those with existing MetS. Thus, healthy diet and lifestyle patternscan be recommended for all people with MetS and should emphasize the consumption of a variety of legumes, cereals (whole grains), fruits, vegetables, fish, and nuts, which have a high nutrient content and are more likely to meet dietary recommendations. This study also raises the possibility that future recommendations and educational campaigns should be most effective in preventing MetS via lifestyle changes.

## Figures and Tables

**Table 1:** Socioeconomicand lifestyle characteristics of participants “with Metabolic Syndrome” (*n* = 333) and “without Metabolic Syndrome” (*n* = 144) stratified by sex.

	Men					Women				
	Without MetS (*n* = 63)		With MetS (*n* = 183)		*p*-Value *	Without MetS (*n* = 81)		With MetS (*n* = 150)		*p*-Value *
	Mean ± SD	Median (IQR)	Mean ± SD	Median (IQR)		Mean ± SD	Median (IQR)	Mean ± SD	Median (IQR)	
Age (y)	63.8 ± 5.9	64.0 (59.0, 67.0)	64.1 ± 5.9	64.0 (59.0, 69.0)	0.544	66.8 ± 5.0	66.0 (63.0, 70.0)	65.9 ± 4.5	66.0(62.0, 69.0)	0.340
BMI (kg/m^2^)	27.0 ± 3.2	27.5 (24.9, 28.7)	32.0 ± 3.6	31.9 (29.0, 34.5)	<0.001	25.3 ± 3.3	25.6 (22.9, 27.4)	32.8 ± 4.2	32.7 (30.1, 36.1)	<0.001
Current smoking habit (%)										
Yes	6.3		14.8		0.081	6.2		12.8		0.119
No	93.7		85.2			93.8		87.2		
Education (%)										
Primary	39.7		37.1		0.660	53.1		60.0		0.595
Secondary	39.7		36.5			30.9		26.9		
University or graduate	20.6		26.4			16.0		13.1		
Total physica lactivity(*n*) ^†^	63		158			81		131		
Total physical activity (MET·hour/week) ^†^	123 ± 208	84 (60, 117)	61 ± 50	46 (24, 85)	<0.001	88 ± 34	84 (63, 107)	60 ± 46	46 (26, 89)	<0.001
MetScomponents										
High blood pressure										
Systolic blood pressure (mmHg)	137.0 ± 19.0	134.5 (124, 143)	141.0 ± 16.9	141 (129.7, 148.5)	0.038	135.9 ± 15.8	136 (125.8, 146.3)	138.4 ± 17.3	137.6 (126.7, 148.6)	0.280
Diastolic blood pressure (mmHg)	81.5 ± 9.4	81.5 (74.5, 88.5)	82.8 ± 9.5	83 (75.7, 89.5)	0.362	79.9 ± 9.0	80.5 (74.3, 86.3)	79.6 ± 9.8	79.7 (74.6, 85.3)	0.828
(%) ^‡^	76.2		95.6		<0.001 ^§^	69.1		88.0		<0.001 ^§^
Hyperglycaemia (mg/dL)	98.3 ± 32.2	97 (71, 119)	119.9 ± 39.0	110 (100, 127)	<0.001	89.0 ± 8.0	89 (83, 94)	110.5 ± 23.4	104 (95, 120)	<0.001
(%) ^‡^	27.0		81.4		<0.001 ^§^	3.7		48.0		<0.001 ^§^
Hypertriglyceridemia (mg/dL)	96.2 ± 9.2	95 (93, 100)	155.6 ± 77.1	133 (96, 198)	<0.001	84.5 ± 27.2	80 (64, 100)	135.3 ± 55.5	125 (91, 169.8)	<0.001
(%) ^‡^	9.5		53.6		<0.001 ^§^	11.0		51.6		<0.001 ^§^
Low HDL-cholesterol (mg/dL)	51.5 ± 9.9	50 (45, 55)	41.2 ± 10.0	40 (35, 46)	<0.001	63.3 ± 11.9	63 (55.5, 71)	49.1 ± 10.7	48 (42, 54.5)	<0.001
(%) ^‡^	11.1		53.0		<0.001 ^§^	22.2		58.0		<0.001 ^§^
Abdominal obesity (cm)	92.9 ± 10.1	94 (87.7, 99.2)	112.1 ± 10.3	111.1 (103.9, 120.5)	<0.001	79.9 ± 7.7	80 (75.4, 85.4)	104.6 ± 11.1	105.5 (97.0, 112.3)	<0.001
(%) ^‡^	12.7		86.3		<0.001 ^§^	11.1		960		<0.001 ^§^

Abbreviations: BMI, body mass index; FA, fatty acids; FQI, fat quality index; IQR, interquartile range; MetS, Metabolic Syndrome; MET, metabolic equivalent of task; MUFAs, monounsaturated fatty acids; PUFAs, polyunsaturated fatty acids; SD, standard deviation; SFAs, saturated fatty acids.* Differences in means between participants without and with MetS were tested by unpaired Students’ *t*-test. ^†^ Participants who did not respond to the physical activity questionnaires were excluded from the analysis (i.e., 25 men and 19 women). ^‡^ Percentage (%) of patients without and with MetS. ^§^ Differences between participants without and with MetS were tested by χ^2^.

**Table 2:** Nutrient intake in participants “with Metabolic Syndrome” (*n* = 333)and “without Metabolic Syndrome” (*n* = 144) stratified by sex.

	Men					Women				
	Without MetS (*n* = 63)		With MetS (*n* = 183)		*p*-Value *	Without MetS (*n* = 81)		With MetS (*n* = 150)		*p*-Value *
	Mean ±SD	Median (IQR)	Mean ±SD	Median (IQR)		Mean ±SD	Median (IQR)	Mean ±SD	Median (IQR)	
Energy intake (kcal/day)	2872 ± 738	2858 (2315, 3282)	2641 ± 689	2561 (2153, 3071)	0.019	2366 ± 698	2323 (1881, 2697)	2071 ± 543	1952 (1713, 2448)	<0.001
Carbohydrate intake (% total E)	44.7 ± 6.2	44.7 (41.3, 48.3)	40.0 ± 6.8	40.7 (34.9, 45.2)	<0.001	44.6 ± 5.2	44.3 (40.7, 47.1)	41.0 ± 6.9	40.9 (36.4, 45.5)	<0.001
Protein intake (% total E)	15.9 ± 2.4	15.6 (14.3, 17.6)	16.3 ± 3.1	15.9 (14.3, 17.7)	0.599	16.9 ± 3.0	16.4 (14.9, 18.5)	18.0± 3.2	18.0 (15.7, 20.4)	0.010
Fat intake (% total E)	36.2 ± 6.1	35.6 (31.6, 40.2)	38.9 ± 7.0	38.6 (34.0, 44.0)	0.008	37.6 ± 5.7	37.8 (32.8, 41.3)	40.9 ± 7.6	40.7 (35.5, 46.1)	<0.001
PUFA (% total E)	7.6 ± 3.4	6.3 (5.3, 9.1)	7.5 ± 3.0	6.7 (5.5, 8.8)	0.673	8.0 ± 3.6	6.6 (5.8, 8.9)	8.1 ± 4.1	6.7 (5.6, 9.2)	0.941
MUFA (% total E)	17.5 ± 4.3	16.8 (14.5, 19.7)	19.3 ± 5.0	18.8 (15.9, 22.2)	0.007	18.9 ± 4.4	18.3 (15.5, 21.1)	21.1 ± 5.9	20.3 (17.1, 24.6)	0.003
SFA (% total E)	11.7 ± 3.5	10.9 (9.6, 12.9)	12.0 ± 3.3	11.4 (9.6, 13.3)	0.517	12.5 ± 3.6	11.6 (9.9, 14.4)	12.5 ± 4.0	11.6 (10.1, 13.8)	0.975
Trans FA (g/d)	8.1 ± 8.9	4.7 (2.9, 7.2)	6.8 ± 7.5	3.8 (2.3, 6.5)	0.123	7.8 ± 8.5	4.9 (2.8, 10.3)	6.4 ± 8.5	3.0 (1.5, 5.4)	0.005
Linoleic acid (g/d)	16.2 ± 10.5	12.5 (8.8, 21.2)	14.5 ± 8.9	11.2 (8.5, 18.8)	0.298	14.7 ± 9.8	11.7 (8.7, 16.8)	12.9 ± 9.6	10.0 (6.5, 16.3)	0.034
ω-3 FA (g/d)	26.0 ± 36.0	9.2 (8.9, 18.9)	21.2 ± 29.8	9.2 (1.2, 18.2)	0.135	26.5 ± 34.5	9.4 (8.7, 35.2)	21.8 ± 34.0	8.9 (1.0, 17.9)	0.003
Linolenic acid (g/d)	7.0 ± 9.0	2.8 (2.5, 5.5)	5.8 ± 7.5	2.8 (0.8, 5.1)	0.168	7.1 ± 8.6	3.1 (2.4, 9.2)	5.8 ± 8.5	2.6 (0.6, 4.9)	0.003
Marine ω-3 FA (g/d)	12.7 ± 18.0	4.4 (4.2, 9.1)	10.3 ± 14.9	4.3 (0.3, 8.9)	0.111	13.0 ± 17.3	4.5 (4.1, 17.4)	10.7 ± 17.0	4.2 (0.3, 8.8)	0.009
Non-marine ω-3 FA (g/d)	13.2 ± 18.0	4.9 (4.5, 10)	10.9 ± 14.9	4.9 (0.9, 9.4)	0.161	13.5 ± 17.3	5.1 (4.5, 17.9)	11.1 ± 17.0	4.6 (0.7, 9.2)	0.002
Animal fat (g/d)	49.8 ± 18.2	46.1 (38.5, 59.7)	48.3 ± 19.6	43.7 (34.8, 59.2)	0.307	41.5 ± 23.1	38.6 (27.3, 50.0)	36.0 ± 13.2	35.0 (26.2, 44.1)	0.091
Vegetable fat (g/d)	65.7 ± 23.4	62.3 (45.2, 85.4)	64.9 ± 22.8	62.8 (48.1, 79.9)	0.799	57.7 ± 19.9	56.1 (41.8, 69.2)	58.3 ± 23.9	56.6 (41.9, 70.4)	0.987
FQI, score	1.9 ± 0.5	1.7 (1.6, 2.1)	2.0 ± 0.4	1.9 (1.7, 2.3)	0.048	1.8 ± 0.4	1.8 (1.6, 2.0)	2.1 ± 0.5	2.0 (1.7, 2.4)	<0.001
Cholesterol (mg/d)	362 ± 105	358 (289, 423)	348 ± 115	334 (274, 399)	0.146	303 ± 122	286 (243, 349)	288 ± 79	283 (250, 355)	0.819
Fiber intake (g/d)	42.2 ± 17.0	38.2 (28.2, 52.0)	32.9 ± 13.1	31.2 (22.6, 39.5)	<0.001	38.6 ± 16.7	34.0 (28.9, 45.3)	31.2 ± 14.9	27.3 (20.9, 36.2)	<0.001

Abbreviations: E, energy; FA, fatty acids; FQI, fat quality index; IQR, interquartile range; MetS, Metabolic Syndrome; MUFAs, monounsaturated fatty acids; PUFAs, polyunsaturated fatty acids; SD, standard deviation; SFAs, saturated fatty acids. * Difference in means between participants without and with MetS were tested by unpaired Students’ *t*-test.

**Table 3:** Food consumption in participants “with Metabolic Syndrome” (*n* = 333) and “without Metabolic Syndrome” (*n* = 144) stratified by sex.

	Men					Women				
	Without MetS (*n* = 63)		With MetS (*n* = 183)		*p*-Value	Without MetS (*n* = 81)		With MetS (*n* = 150)		*p*-Value *
	Mean ±SD	Median (IQR)	Mean ±SD	Median (IQR)		Mean ±SD	Median (IQR)	Mean ±SD	Median (IQR)	
Fruits (g/day)	487 ± 205	495 (344, 627)	402 ± 229	364 (220, 546)	0.002	576 ± 218	553 (419, 697)	394 ± 214	352 (242, 499)	<0.001
Vegetables (g/day)	346 ± 147	341 (232, 426)	311 ± 157	284 (192, 415)	0.075	357 ± 151	334 (258, 431)	343 ± 159	327 (242, 420)	0.407
Potatoes (g/day)	96.7 ± 45.8	95.7 (57.1, 149.8)	70.2 ± 45.2	56.0 (31.4, 97.4)	<0.001	77.6 ± 45.0	85.7 (38.6, 107.1)	67.3 ± 57.9	49.5 (28.0, 94.1)	0.013
Legumes (g/day)	20.5 ± 14.7	16.6 (12.0, 25.1)	18.9 ± 12.9	16.1 (12.1, 24.8)	0.901	18.0 ± 12.2	16.0 (12.0, 21.1)	17.8 ± 12.3	16.1 (12.0, 21.6)	0.582
Olives and EVOO (g/day)	34.7 ± 34.0	28.3 (10.0, 46.4)	39.3 ± 28.2	32.0 (21.0, 50.0)	0.070	24.7 ± 16.5	25.0 (12.4, 32.1)	29.8 ± 24.0	28.3 (10.9, 46.0)	0.289
Other olives oils	14.3 ± 16.7	10.0 (0.0, 25.0)	13.0 ± 16.4	4.2 (0.0, 25.0)	0.563	15.9 ± 15.4	10.0 (0.0, 25.0)	15.1 ± 14.8	10.0 (0.0, 25.0)	0.724
Other oils and fats	4.4 ±9.2	1.3 (0.0, 4.3)	4.9± 8.9	0.8 (0.0, 5.0)	0.856	4.7± 6.8	2.1 (0.7, 5.8)	3.9 ± 6.6	0.8 (0.0, 5.0)	0.112
Nuts (g/day)	15.8 ± 17.3	8.6 (4.0, 25.7)	13.3 ± 13.3	8.4 (4.0, 21.0)	0.594	14.7 ± 13.6	8.6 (4.3, 25.7)	11.7 ± 13.5	7.2 (2.0, 16.7)	0.023
Totalfish (g/day)	96.3 ± 36.2	88.1 (68.1, 120.5)	87.7 ± 45.2	80.3 (56.6, 111.3)	0.049	87.4 ± 37.5	80.7 (60.3, 107.4)	88.1 ± 42.2	80.7 (56.6, 115.1)	0.925
White fish	25.4 ± 19.7	21 (10, 21)	26.3 ± 22.4	21.0 (10.1, 21.4)	0.620	28.0 ± 21.6	21.4 (10.0, 42.9)	28.3 ± 22.9	21.0 (10.1, 63.0)	0.362
Bluefish	21.9 ± 19.9	18.6 (8.7, 18.6)	17.2 ± 16.8	8.7 (8.7, 18.2)	0.121	18.1 ± 17.0	18.6 (8.7, 18.6)	20.2 ± 18.7	18.2 (8.7, 18.6)	0.769
Seafood	35.6 ± 14.6	30.7 (26.7, 45.9)	31.1 ± 23.7	30.8 (17.4, 35.2)	0.096	31.6 ± 17.8	30.7 (26.7, 33.0)	28.9 ± 22.3	30.7 (13.4, 31.9)	0.985
Canned fish/seafood	11.7 ± 10.5	7.1 (3.3, 21.4)	11.0 ± 9.6	7.0 (3.4, 21.0)	0.215	8.3 ± 7.4	6.7 (3.3, 12.4)	9.4 ± 8.5	7.0 (3.4, 13.0)	0.115
Total cereal (g/day)	229.3 ± 131.7	222.8 (131.4, 251.6)	159 ± 89	135.9 (91.8, 217.7)	<0.001	149 ± 82	126 (95, 222)	122.8± 69.8	102.4 (79.7, 164.3)	0.004
Whole grain bread	105.2 ± 122.3	75.0 (5.0, 187.5)	61.4 ± 73.8	31.5 (5.0, 75.0)	0.012	66.7 ± 60.0	75.0 (32.1, 75.0)	57.1 ± 63.5	31.5 (5.0, 75.0)	0.019
Refined grain bread	85.3 ± 108.0	32.1 (5.0, 187.5)	66.6 ± 83.1	31.5 (5.0, 75.0)	0.329	47.2 ± 71.1	10.7 (0.0, 75.0)	39.3 ± 53.6	31.5 (0.0, 75.0)	0.895
Rice and pasta	34.5 ± 14.7	34.3 (17.1, 51.4)	27.6 ± 18.2	25.2 (12.4, 34.3)	<0.001	28.7 ± 17.5	17.1 (17.1, 34.3)	23.1 ± 15.1	17.0 (12.4, 33.6)	0.001
Total dairy products (g/day)	295 ± 168	289 (215, 342)	303 ± 216	269 (181, 363)	0.612	312 ± 214	282 (150, 394)	264 ± 164	246 (148, 342)	0.131
Dairy esserts	31.9 ± 34.6	15.3 (6.7, 51.2)	33.6 ± 47.2	15.3 (6.7, 43.0)	0.930	19.9 ± 27.9	8.7 (6.7, 24.1)	18.4± 27.9	6.7 (0.0, 23.4)	0.814
Cheese	32.9 ± 26.9	24.8 (21.4, 44.5)	32.1 ± 31.2	24.4 (14.0, 42.9)	0.395	33.1 ± 23.2	28.1 (19.6, 48.0)	29.3 ± 22.3	24.4 (10.4, 42.0)	0.179
Skimmed dairy	84.8 ± 137.6	8.3 (0.0, 125.0)	115 ± 202	52.5 (0.0, 156.0)	0.215	141.6 ± 194.8	53.6 (0.0, 209.5)	114.4± 136.5	52.5 (0.0, 200.0)	0.529
Whole-fat dairy	144.1 ± 141.3	125.0 (8.3, 208.3)	120 ± 141	84.0 (0.0, 200.0)	0.090	112.5 ± 158.6	17.9 (0.0, 200.0)	100 ± 140	17.5 (0.0, 200.0)	0.765
Total meat (g/day)	152.0 ± 61.1	137 (112, 202)	166.2 ± 71.7	154 (117, 204)	0.247	130 ± 61.7	118 (93, 165)	140 ± 56.6	139 (104, 172)	0.076
Processed meat	40.7 ± 27.0	34.0 (27.0, 52.0)	46.9 ± 34.7	39.1 (21.0, 62.0)	0.433	31.4 ± 21.4	30.0 (18.2, 39.5)	34.0 ± 28.3	28.7 (16.7, 42.7)	0.869
Other meats,	108.4 ± 48.8	104.3 (71.4, 135.7)	116 ± 57	107 (76, 149)	0.500	97.2 ± 51.6	87.6 (64.8, 122.9)	103.8± 46.3	103.7 (74.9, 135.8)	0.172
Bakery godos (g/day)	60.4 ± 44.5	51.2 (26.7, 72.4)	52.1 ± 45.0	44.5 (20.6, 66.7)	0.101	51.0 ± 30.2	46.5 (26.5, 74.2)	37.2 ± 30.6	31.0 (10.4, 53.7)	<0.001
Ready-to-eat-meals	35.0 ±34.2	26.2 (13.6, 37.6)	27.8 ± 40.3	15.4 (9.4, 30.0)	0.003	19.9 ± 18.7	15.3 (4.3, 26.2)	20.5 ± 23.3	15.4 (2.0, 26.4)	0.357
Alcohol (g/day)	230 ± 183	198 (82, 337)	291 ± 322	200 (76, 367)	0.753	109 ± 128	47.1 (0.0, 170)	70 ± 101	28.8 (0.0, 100.0)	0.032

Abbreviations: EVOO, extra virgin olive oil; IQR, interquartile range; MetS, Metabolic Syndrome; SD, standard deviation. * Difference in means between participants without and with MetS were tested by unpaired Students’ *t*-test.

**Table 4:** Percentage of participants “with Metabolic Syndrome” and “without Metabolic Syndrome” below, inside, and above Acceptable Macronutrient Distribution Range (AMDR) proposed by the Institute of Medicine.

Variable	AMDR	Group	% below	% inside	% above	p *
All						
Carbohydrate	45–65%	Without MetS	55.6	44.4	0.0	<0.001
		With MetS	72.7	27.3	0.0	
Protein	10–35%	Without MetS	0.0	100.0	0.0	0.510
		With MetS	0.3	99.7	0.0	
Total fat	20–35%	Without MetS	0.0	39.6	60.4	0.001
		With MetS	0.0	24.9	75.1	
MUFAs	>20%	Without MetS	72.2	-	27.8	0.001
		With MetS	55.6	-	44.4	
LA	5–10%	Without MetS	66.7	20.1	13.2	0.159
		With MetS	64.9	26.4	8.7	
ALA	0.6–1.2%	Without MetS	21.5	29.2	49.3	0.005
		With MetS	36.3	21.0	42.6	
Men						
Carbohydrate	45–65%	Without MetS	52.4	47.6	0.0	0.003
		With MetS	72.7	27.3	0.0	
Protein	10–35%	Without MetS	0.0	100.0	0.0	0.557
		With MetS	0.5	99.5	0.0	
Total fat	20–35%	Without MetS	0.0	46.0	54.0	0.008
		With MetS	0.0	27.9	72.1	
MUFAs	>20%	Without MetS	77.8	-	22.2	0.025
		With MetS	62.3	-	37.7	
LA	5–10%	Without MetS	65.1	22.2	12.7	0.276
		With MetS	66.7	26.8	6.6	
ALA	0.6–1.2%	Without MetS	22.2	38.1	39.7	0.119
		With MetS	35.0	27.3	37.7	
Women						
Carbohydrate	45–65%	Without MetS	58.0	42.0	0.0	0.023
		With MetS	72.7	27.3	0.0	
Protein	10–35%	Without MetS	0.0	100.0	0.0	1.000
		With MetS	0.0	100.0	0.0	
Total fat	20–35%	Without MetS	0.0	34.6	65.4	0.029
		With MetS	0.0	21.3	78.7	
MUFAs	>20%	Without MetS	67.9	-	32.1	0.003
		With MetS	47.3	-	52.7	
LA	5–10%	Without MetS	67.9	18.5	13.6	0.427
		With MetS	62.7	26.0	11.3	
ALA	0.6–1.2%	Without MetS	21.0	22.2	56.8	0.019
		With Met	38.0	13.3	48.7	

Abbreviations: ALA, α-linolenic acid; LA, linoleic acid; MetS, metabolic syndrome; MUFAs, monounsaturated fatty acids. * The differences in prevalence across the two comparison groups was examined using χ^2^.

**Table 5:** Percentage of participants “with Metabolic Syndrome” and “without Metabolic Syndrome” below, inside, and above the 2020 Nutritional Objectives for the Spanish Population proposed by the Spanish Society of Community Nutrition.

Variable	Nutritional Objectives	Group	% Below	% Inside	% Above	p *
Carbohydrate	50–55%	Without MetS	83.3	12.5	4.2	0.004
		With MetS	93.1	5.7	1.2	
Protein	10–20%	Without MetS	0.0	100.0	0.0	1.000
		With MetS	0.0	100.0	0.0	
Total fat	30–35%	Without MetS	9.7	29.9	60.4	0.001
		With MetS	9.3	15.6	75.1	
MUFAs	20%	Without MetS	72.2	-	27.8	0.001
		With MetS	55.6	-	44.4	
PUFAs	5%	Without MetS	14.6	-	85.4	0.774
		With MetS	15.6	-	84.4	
LA	3%	Without MetS	18.1	-	81.9	0.217
		With MetS	23.1	-	76.9	
ALA	1–2%	Without MetS	42.4	30.6	27.1	0.417
		With MetS	48.6	28.5	22.8	
SFA	7–8%	Without MetS	2.8	3.5	93.8	0.952
		With MetS	3.3	3.6	93.1	
Trans FA	<1%	Without MetS	21.5	-	78.5	0.001
		With MetS	36.6	-	63.4	
DHA	300 mg	Without MetS	100.0	-	0.0	1.000
		With MetS	100.0	-	0.0	
Total fiber	M: 35 g/d	Without MetS	27.8	-	72.2	<0.001
	F: 25 g/d	With MetS	52.0	-	48.0	
Cholesterol	<300 mg/d	Without MetS	41.0	-	59.0	0.123
		With MetS	48.6	-	51.4	
Fruits	>300 g/d	Without MetS	11.1	-	88.9	<0.001
		With MetS	35.4	-	64.6	
Vegetables	>250g/d	Without MetS	24.3	-	75.7	0.032
		With MetS	34.2	-	65.8	
Sugar foods	<6%	Without MetS	0.7	-	99.3	0.905
		With MetS	0.6	-	99.4	

Abbreviations: ALA, α-linolenic acid; DHA, docosahexaenoic acid; FA, fatty acid; LA, linoleic acid; MetS, metabolic syndrome; MUFA, monounsaturated fatty acids; PUFAs, polyunsaturated fatty acids; SFAs, saturated fatty acids. * Differences in prevalence between groups were assessed by χ^2^.
